# Information-seeking behaviors and barriers to the incorporation of scientific evidence into clinical practice: A survey with Brazilian dentists

**DOI:** 10.1371/journal.pone.0249260

**Published:** 2021-03-25

**Authors:** Branca Heloisa Oliveira, Izabel Monteiro D. Hyppolito, Zilson Malheiros, Bernal Stewart, Claudio Mendes Pannuti

**Affiliations:** 1 Faculty of Dentistry, Department of Community and Preventive Dentistry, Rio de Janeiro State University, Rio de Janeiro, Rio de Janeiro, Brazil; 2 Latin American Oral Health Association (LAOHA), São Paulo, Brazil; 3 Colgate Palmolive Company, Global Technology Center, Piscataway, NJ, United States of America; 4 Department of Stomatology, School of Dentistry, University of São Paulo, São Paulo, Brazil; Imam Abdulrahman Bin Faisal University, SAUDI ARABIA

## Abstract

The aim of this online cross-sectional study is to identify the sources of scientific information used by Brazilian dentists in clinical decision-making and the barriers that they perceive as important to the incorporation of scientific evidence into clinical practice. A pretested questionnaire created in Google Forms which was made available to participants through links sent by e-mail or shared on Facebook® and Instagram® was used to collect the data between October 2018 and May 2019. Only dentists who were involved in direct or indirect care of patients (i.e. clinicians who performed dental procedures or dental educators who participated in the clinical training of graduate or postgraduate dental students) were asked to complete the questionnaire. The sample was comprised of 528 dentists (the response rate from the alumni database was 6.9%); their mean age was 45.2 years (±12.5) and 30.9% had an academic position. The majority were women (68.0%) and lived in Southern or Southeastern Brazil (96.0%). The sources of scientific information more frequently used by them in clinical decision-making were clinical guidelines (65.1%; 95% CI: 60.9, 69.2), scientific articles (56.8%; 95%CI: 52.5, 61.1) and bibliographic databases (48.3%; 95% CI: 43.9, 52.6). The information resource less frequently used was social media. The most important barriers to the clinical use of scientific evidence were: difficulty in determining whether scientific contents found on the Internet were reliable or not (41.8%; 95% CI: 37.6, 46.2), high cost of access to scientific papers (37.7%; 95% CI: 33.5, 41.9), and lack of time for reading scientific articles (32.4%; 95% CI: 28.4, 36.6). Although Brazilian dentists show a positive attitude towards obtaining scientific evidence from reliable sources, there still remain important barriers to the translation of evidence into practice. This can have significant implications for quality of care and should be further investigated.

## Introduction

Incorporating scientific evidence into dental practice requires that dentists apply the best available scientific evidence that may be relevant to a given patient case in the process of clinical decision-making [1]. However, oral health care providers need to be skilled in locating and critically appraising reports of scientific studies to find such evidence [2]. Moreover, clinicians and dental educators should become lifelong learners and seek to actively participate in closing the knowledge gaps in clinical dentistry [1].

The process by which evidence produced by scientific research is made available for use in clinical and political decisions is called knowledge transfer and exchange (KTE). Unfortunately, there are numerous barriers to KTE, at both the organizational and individual levels. For example, at the organizational level, the professional incentive system for scientists encourages the dissemination of results of scientific studies in high-impact peer-reviewed journals, which are generally published in the English language. This can make it difficult for clinicians in countries where a language other than English is spoken to access updated scientific information in their field. At the individual level, the lack of ability to evaluate the results of scientific studies, negative attitudes towards change and conflicts of interest are often considered as important barriers to the successful transfer of knowledge between producers and users of scientific evidence [3].

Additionally, it is very challenging for dentists to keep up to date with the latest evidence because of the current scenario of large dental research productivity. A total of 104,975 articles were published between 2009 and 2019 in dental journals and indexed by MEDLINE. In view of these numbers, feeling overwhelmed with information and having difficulty in selecting evidence that may be truly relevant and useful for clinical practice are not unlikely [4].

The identification of dentists’ behaviors regarding the search for scientific information as well as barriers they face in this process is an indispensable step for the development of effective KTE strategies. In developed countries, it has been shown that dentists are cautious about making decisions based on documentary sources like systematic reviews of the dental literature and prefer to seek advice from colleagues, dental specialists, or respected dental experts. They also report lacking time, experience, skills, and confidence to find and use evidence-based resources [5]. Moreover, in a recently published systematic review, it was shown that the barriers most frequently reported by dentists in the application of evidence-based principles in their practice were lack of time, financial constraints and inability to find and appraise scientific articles [6].

Knowledge about the frequency of use of scientific information resources by Brazilian dentists is scarce, and data regarding the use of clinical guidelines and systematic reviews published in the Cochrane Library are lacking. Furthermore, some potential constraints which may hinder the use of scientific evidence in clinical practice such as the high cost of accessing peer-reviewed papers, difficulty in performing critical appraisal of research articles, and uncertainty regarding the quality of the information found on the Internet have not been investigated yet [7]. Thus, the main objective of this study was to identify the frequency by which Brazilian dentists use various resources in order to obtain scientific information for clinical decision-making and to assess their perceived importance of some prespecified barriers to the incorporation of scientific evidence into clinical practice. Moreover, we were interested in identifying factors that might influence the use of the Cochrane Library and the perception of the difficulty in performing critical appraisal of evidence as a barrier to its incorporation into practice. Therefore, we sought to assess the relationship between age, sex, year of graduation, and academic position and these outcomes.

## Material and methods

The Institutional Review Board of the Pedro Ernesto Hospital of Rio de Janeiro State University (UERJ) approved this cross-sectional study before its outset (CAAE 94336518.9.0000.5259). Data were collected online, and before answering the questionnaire, all participants were requested to click a button stating that they agreed to participate in the study. The reporting follows the STROBE guideline for cross-sectional studies [8] and SURGE [9].

### Participants

The participants were alumni of a major Brazilian public university (USP, University of São Paulo, Brazil), and dentists recruited via social media (i.e., Facebook® or Instagram®). Any Brazilian dentist was considered eligible for the study; there were no exclusion criteria such as age, sex, or graduation year.

In order to contact USP alumni we sent e-mails to 5,990 dentists who had at some point enrolled in graduate or postgraduate programs offered by its School of Dentistry. We invited them to participate in our survey regarding the use of scientific evidence in clinical decision-making and provided a link to a Google Form containing the questionnaire. We also asked them to help us with the recruitment of participants by forwarding our e-mail to at least two colleagues who they thought might be willing to participate in the study. After the first email, two reminders were sent to all potential participants.

Additionally, to promote the study on social media, the researchers used their personal accounts on Facebook® and Instagram®. Also, a business account managed by a group of professors of another major Brazilian public university (https://pt-br.facebook.com/crescersorrindo), was used for this purpose. The publications on social media regarding the research provided a link to a Google Form containing the questionnaire.

These strategies were used in order to recruit as many participants as possible, since we were not able to obtain a complete list of electronic addresses of all registered Brazilian dentists.

The primary outcome considered for sample size calculation was the percentage of dentists that would report using the Cochrane Library, often or very often, in order to obtain scientific information for clinical decision-making. Considering that we had no information from previous studies regarding how varied the population of Brazilian dentists is with respect to the use of this source of information, we adopted a conservative approach. Thus, we estimated that we would need to recruit at least 384 participants to allow for the calculation of a 95% confidence interval for the expected frequency of 50% of dentists using the Cochrane Library, with an error margin of no more than 0.05. No correction for finite population was used since the number of registered dentists in Brazil is very large (i.e., more than 30,000). We used the formula “n = 100 + 50(i)”, where (i) refers to number of independent variables in the final model, to verify whether this sample size would be sufficient for performing logistic regression analyses with four explanatory variables [10]. The calculation resulted in a sample of 300 individuals, which was smaller than our target sample of 384 participants.

### Questionnaire

An online questionnaire created in Google Forms (Google Inc., Mountain View, CA, USA) was used to collect the data. The questions and responses were developed during directed group discussions. The list of questions to be asked was drawn up by four members of the research team (BHO, ZM, BS, and CMP), taking into consideration the objectives of the study and the target population. The sources of acquisition of information for clinical decision-making were based on their own experience and the dental literature [11–13]. Barriers to the use of scientific evidence in clinical practice were selected from those already identified in previous studies [3,5,14]. Questions, responses, and instructions to the respondents were written and organized focusing on minimizing respondent burden [15].

From May to June 2018, we pretested the questionnaire with 34 dentists who were postgraduate students at the University of São Paulo (n = 21) and the Rio de Janeiro State University (n = 13). Following the pretest, the research team made revisions to the original document regarding wording, sequence of questions, and response options in order to ensure that the questionnaire presented adequate content validity. The final questionnaire was organized into five sections. In the first section, we explained the aims of the study, introduced the researchers responsible for its development, invited potential participants to answer the questionnaire, and requested those who were willing to participate in the survey to click a button stating that they voluntarily agreed to participate. In the second section, we asked respondents about their year of birth, sex, place of residence, and graduation year. We also asked whether or not they were involved in direct (i.e., performed dental procedures) or indirect (i.e., participated in the clinical training of graduate or postgraduate dental students) care of patients. We requested dentists who did not perform any kind of clinical work to stop filling out the questionnaire at this point. The third section was comprised of questions about respondents’ professional activity. The fourth section included questions about the frequency of use of the following sources of scientific information in the process of clinical decision-making, during the past 12 months: consulting with colleagues, textbooks, clinical guidelines, bibliographic databases (e.g., MEDLINE/PubMed, Embase, and Lilacs), scientific articles, the Cochrane Library, search engines (e.g., Google®, Yahoo® and Bing®), and social media (e.g., Facebook®, Instagram® and YouTube®). The answers to these questions were given on a five‐point frequency scale: never, hardly ever, occasionally, often or very often. The wording of this scale was the same as the one used in the Brazilian version of the Oral Health Impact Profile-Short Form (OHIP-14) [16]. The fifth section included questions about barriers to the use of scientific evidence in clinical practice that they might have experienced during the past 12 months: lack of time to read scientific papers, high cost of access to scientific papers, insufficient proficiency in the English language to read and understand the contents of scientific papers, lack of confidence to critically appraise evidence from scientific journal articles, and difficulty in determining whether scientific contents found on the Internet were reliable or not. Response options were given on a 5‐point Likert‐scale that ranged from 1 (strongly disagree) to 5 (strongly agree). The full questionnaire in Portuguese (S1 Appendix) and English (S2 Appendix) is available as supporting information.

Data collection lasted from October 2018 to April 2019 for USP alumni and from March to May 2019 for social media.

### Statistical analysis

Descriptive analyses were performed, and distributions of absolute and relative frequencies (percentages with 95% confidence intervals) were obtained.

In order to estimate the percentage of dentists (with 95% confidence interval) who used each source of information, often or very often, in the past 12 months, responses were recoded as follows: “often” and “very often” as “yes” (i.e., used the source of information) and “never”, “hardly ever” and “occasionally” as “no” (i.e., did not use the source of information). In order to estimate the percentage of dentists (with 95% confidence interval) who perceived lack of time, cost of access to scientific papers, insufficient proficiency in the English, lack of confidence to critically appraise evidence, and difficulty in determining reliability of Internet contents as barriers to the use of evidence in practice, their responses were also recoded by grouping “strongly agree” and “agree” into “yes” (i.e., perceived the factor as a barrier to the use of scientific evidence in clinical practice) and “strongly disagree”, “disagree” and “neither agree nor disagree” into “no” (i.e., did not perceive the factor as a barrier to the use of scientific evidence in clinical practice).

Student’s t-tests were used to analyze whether there were significant differences between the mean age of those (1) who used the Cochrane Library and those who did not and (2) who perceived difficulty in performing critical appraisal as a barrier to the incorporation of evidence into practice and those who did not. The significance level was set at α = 0.05. Additionally, we performed logistic regression to evaluate the relationship between these outcome variables and age, sex, graduation year and academic position. To select variables for the multivariate models, the *p*-value cut-off point of 0.25 in the bivariate analyses was used for [17]. All analyses were performed using Stata 14.0 software (StataCorp, College Station, TX, USA).

## Results

We received 412 responses from dentists contacted through the university’s alumni database and 155 responses from dentists contacted through social media making up a total of 567 responses. Among the 567 respondents, one subject from the USP subsample checked “no” in the box corresponding to the question asking for consent to participate in the study. This questionnaire was excluded and the initial sample totaled 566 respondents. Thus, the response rate from the USP alumni database was 6.9%. We could not calculate overall response rate because the number of dentists who actually received the invitations to participate in the survey via social media was unknown. Thirty-eight of the 566 respondents mentioned that they did not provide clinical care to dental patients, either directly or indirectly. Thus, they did not respond to the questions regarding sources of scientific information used for clinical decision-making and barriers to the use of scientific evidence in clinical practice and we ended up with 528 participants. The age range of the participants was from 23 to 83 years, and the majority lived in Southern or Southeastern Brazil (96.0%), were women (68.0%), and did not hold an academic position (69.1%). Detailed information on sociodemographic characteristics of the study population is depicted in Table 1.

**Table 1:** Distribution of respondents (n = 528) according to sociodemographic characteristics and source of recruitment.

	Source of participants’ recruitment		Total
	University’s alumni database	Social media	
Characteristics	n = 379 (71.8) n (%)	n = 149 (28.2) n (%)	N = 528 (100.0) N (%)
**Place of residence (by macroregion)**			
Southeast or South	369 (97.4)	138 (92.6)	507 (96.0)
North, Northeast or Midwest	10 (2.6)	11 (7.4)	21 (4.0)
**Graduation year (by decade)**			
≤ 1980	39 (10.3)	6 (4.0)	45 (8.5)
≥ 1981 and ≤ 1990	96 (25.3)	23 (15.4)	119 (22.5)
≥ 1991 and ≤ 2000	89 (23.5)	49 (32.9)	138 (26.2)
≥ 2001 and ≤ 2010	78 (20.6)	39 (26.2)	117 (22.2)
≥ 2011	77 (20.3)	32 (21.5)	109 (20.6)
**Sex**			
Female	235 (62.0)	124 (83.2)	359 (68.0)
Male	142 (37.5)	25 (16.8)	167 (31.6)
Opt not to indicate	2 (0.5)	0	2 (0.4)
**Academic position**			
Yes	117 (30.9)	46 (30.9)	163 (30.9)
No	262 (69.1)	103 (69.1)	365 (69.1)
**Age (years)**			
Mean (SD)	46.7 (12.9)	43.1 (10.4)	45.2 (12.5)

SD: Standard deviation.

**Fig 1:**
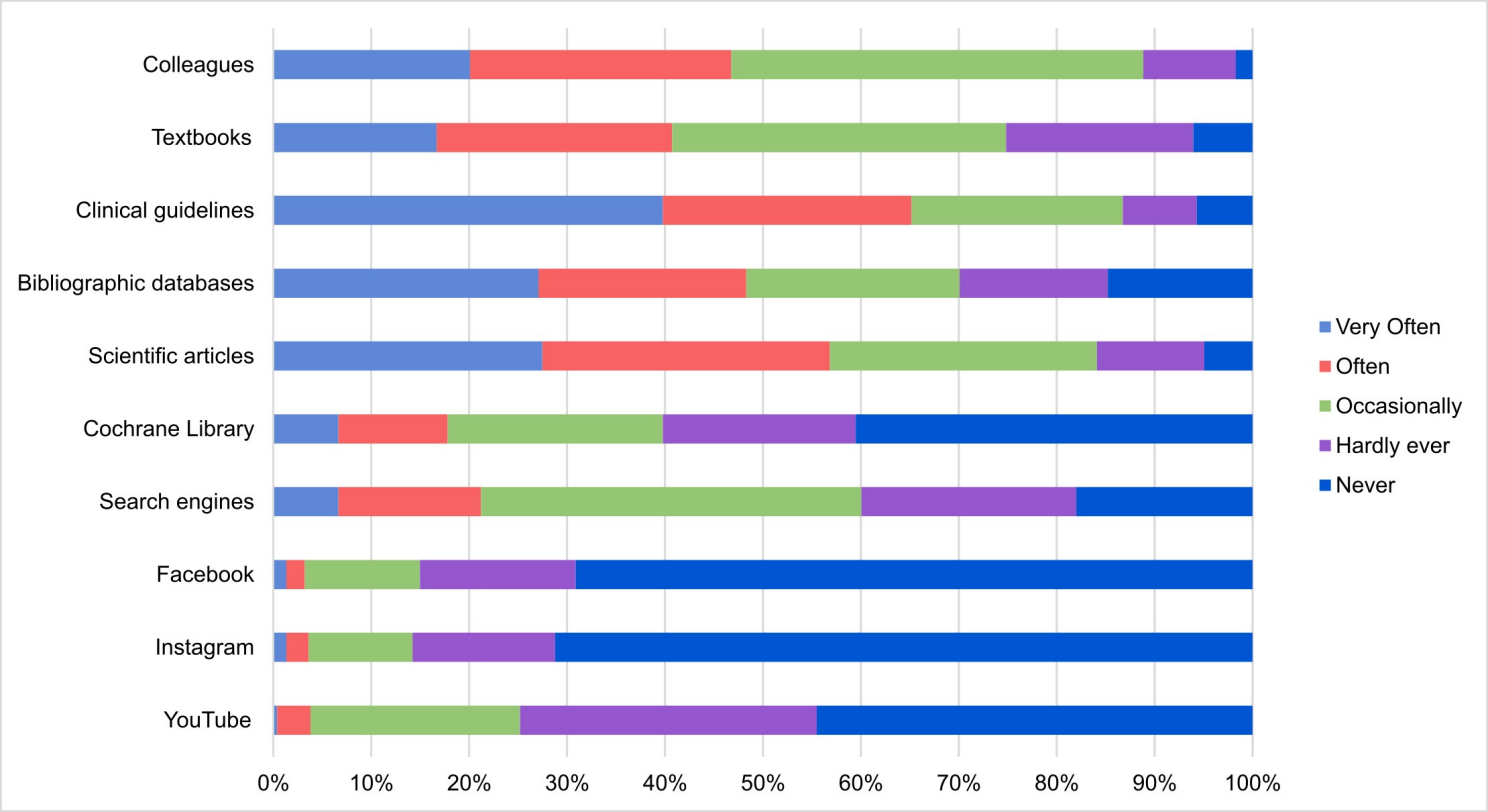
Relative frequency of use (very often, often, occasionally, hardly ever or never) of different sources of scientific information for clinical decision-making by study participants (N = 528) in the past 12 months.

The source of scientific information more frequently used by respondents was clinical guidelines, followed by scientific articles and bibliographic databases. The information resources less frequently used were Facebook®, Instagram®, and YouTube® (Fig 1 and Table 2).

**Table 2:** Relative frequency (proportion, 95% confidence interval, CI) of use of different sources of scientific information for clinical decision-making and relative frequency of perceived barriers to the use of scientific information in clinical practice in the past 12 months by study participants (N = 528).

**Sources of scientific information**	**Relative frequency (often or very often)**	**95% CI**
Consulting with colleagues	46.8	42.4, 51.1
Textbooks	40.7	36.5, 45.0
Clinical guidelines	65.1	60.9, 69.2
Bibliographic databases	48.3	43.9, 52.6
Scientific articles	56.8	52.5, 61.1
Cochrane Library	17.8	14.6, 21.3
Search engines	21.2	17.8, 24.9
Social media		
Facebook	3.2	1.9, 5.1
Instagram	3.6	2.1, 5.5
YouTube	3.7	2.3, 5.7
**Perceived barriers to the use of scientific information**	**Relative frequency (strongly agree or agree)**	**95% CI**
Lack of time to read papers	32.4	28.4, 36.6
High cost of papers	37.7	33.5, 41.9
Insufficient proficiency in English	23.5	19.9, 27.3
Lack of confidence to perform critical appraisal	25.2	21.5, 29.1
Difficulty in determining reliability of Internet contents	41.8	37.6, 46.2

**Fig 2:**
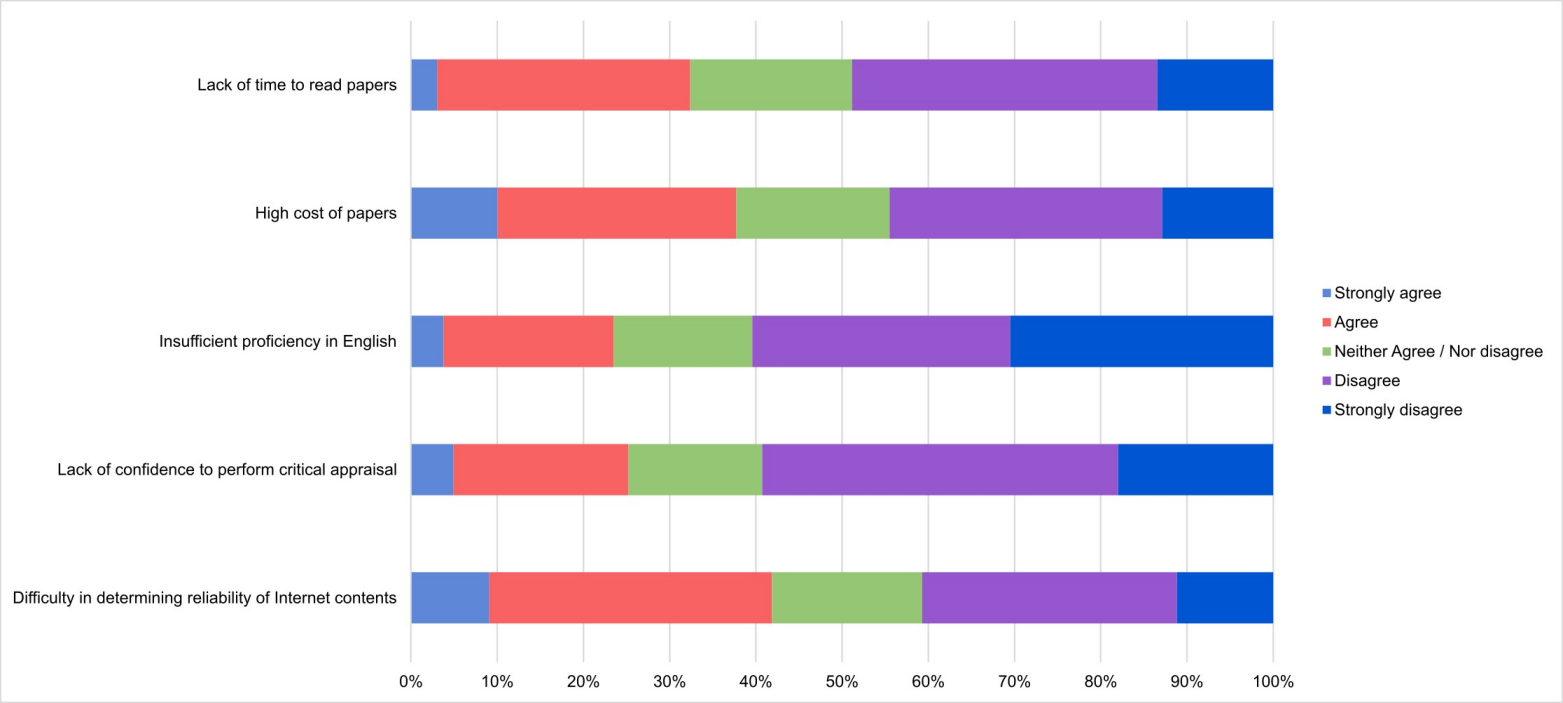
Relative frequency of agreement (strongly agree, agree, neither agree nor disagree, disagree, strongly disagree) to different factors perceived as barriers to the use of scientific information in clinical practice in the past 12 months by study participants (N = 528).

Based on the participants’ experience during the past 12 months, difficulty in determining whether scientific contents found on the Internet were reliable or not and high cost of access to scientific papers, were the barriers to the use of scientific information in clinical practice more frequently perceived by them, followed by lack of time for reading scientific articles. Insufficient proficiency in English was the problem less frequently reported (Fig 2 and Table 2).

The Cochrane Library was reported to be used often or very often by 17.8% of respondents (Table 2). The odds of reporting the use of the Cochrane Library was 3.6 times higher in respondents who held an academic position compared to those who did not hold an academic position (Table 3).

**Table 3:** Logistic regression analyses of the use of the Cochrane Library as a source of scientific information and difficulty in critically appraising scientific studies as a barrier to the incorporation of evidence into practice, with age, sex, graduation year, and academic position.

**Variable**		**Odds ratio of use of the Cochrane Library as a source of scientific information**					
		**Unadjusted model**			**Adjusted model**		
		OR	95% CI	p-value	OR	95% CI	p-value
**Graduation year**	≤ 1980	1					
	≥ 1981 and ≤ 1990	1.01	0.37, 2.77	0.98	-	-	-
	≥ 1991 and ≤ 2000	1.58	0.61, 4.12	0.35	-	-	-
	≥ 2001 and ≤ 2010	2.04	0.78, 5.33	0.14	-	-	-
	≥ 2011	1.20	0.44, 3.27	0.72	-	-	-
**Sex**	Male	1					
	Female	1.12	0.69, 1.82	0.65	-	-	-
**Academic position**	No	1					
	Yes	3.60	2.27, 5.71	0.00	-	-	-
**Age**		0.99	0.97, 1.01	0.28	-	-	-
**Variable**	**Odds ratio of perceiving difficulty in critically appraising scientific evidence as a barrier to its incorporation into practice**					
	**Unadjusted model**			**Adjusted model**		
	OR	95% CI	p-value	OR	95% CI	p-value
**Graduation year**	≤ 1980	1			1		
	≥ 1981 and ≤ 1990	1.59	0.64, 3.97	0.32	-	-	-
	≥ 1991 and ≤ 2000	1.64	0.67, 4.02	0.28	-	-	-
	≥ 2001 and ≤ 2010	2.04	0.83, 5.04	0.12	-	-	-
	≥ 2011	2.57	1.04, 6.32	0.04	-	-	-
**Sex**	Male	1			1		
	Female	1.57	1.00, 2.45	0.05	1.40	0.89, 2.23	0.15
**Academic position**	No	1			1		
	Yes	0.99	0.65, 1.53	0.99	-	-	-
**Age**		0.98	0.96, 0.99	0.02	0.98	0.97, 1.00	0.06

Lack of confidence to critically appraise evidence from scientific journal articles was perceived as a barrier to the use of evidence in the process of clinical decision-making by 25.2% of respondents (Table 2). Respondents who agreed that this could be a problem were significantly younger than those who did not (43.1 years and 45.9 years, respectively. *t*-test, *p* = 0.02). Additionally, the odds of perceiving difficulty in performing critical appraisal as a barrier to the use of scientific evidence in practice was 57% higher for female than for male participants. After adjusting for age, this association was no longer significant (Table 3).

## Discussion

The resources more often used in the process of clinical decision-making by Brazilian dentists who participated in our study were clinical guidelines, scientific articles and bibliographic databases (e.g., MEDLINE/PubMed, Embase and Lilacs) were. Also, practices widely reported were consulting with colleagues and looking for information on textbooks. Participants considered concerns regarding reliability of online scientific information, financial constraints for accessing scientific papers, and shortage of time for reading scientific papers relevant hindrances to integrating evidence into practice.

Evidence-based clinical guidelines (CGs) are important tools for reducing the gap between research and clinical practice and consequently improving the outcomes of dental care [18]. Systematic reviews, which can be arduous to read and comprehend by most health care professionals, are the basis for high quality CGs. The development of CGs follows several steps and should include some key components, such as the use of systematic evidence review methods, rating of quality and reliability of evidence, disclosure of financial and nonfinancial conflicts of interest, and clear recommendations regarding benefits, harms, and possibly costs [18,19]. Moreover, CGs must be prepared in a clinician-friendly format and language [1].

The dentists in our sample showed a positive attitude towards CGs. High acceptability of CGs was also shown in a survey with European dentists who acknowledged the multidimensional benefits of CGs to both clinicians and patients and suggested that CGs should be more effectively disseminated through partnerships between National Dental Associations and Universities [20]. However, the potential benefits of guidelines are as good as the quality of the guidelines themselves [21]. Although the quality of dental CGs has been improving in the past years, it is still suboptimal and wide variation in the overall quality of guidelines produced by different dental specialties exists [22].

The high rate of use of bibliographic databases suggests that our surveyed dentists understand that these are important resources for finding peer-reviewed scientific articles. Nevertheless, efficiently navigating electronic databases may be a daunting task [2]. Furthermore, as science is increasingly published in English, non-English speakers may suffer a genuine disadvantage [23]. Overall English proficiency levels in Brazil are very low, with only around 5% of Brazilians stating that they have some knowledge of English [24]. In our study, approximately one in four participants agreed that insufficient proficiency in English was a barrier to the understanding of scientific articles. The language barrier has also been shown to negatively affect evidence-based practice among Brazilian physical therapists [25]. These findings indicate that although the adoption of English as the “universal language of science” has allowed scientists to communicate globally, the primary use of a single language in science communication may prevent universal and equitable access to scientific knowledge [26].

Additionally, clinicians may be able to read in English but they still may not master the competencies required to evaluate the quality of published articles before implementing a new technique or changing his/her clinical decision-making [27]. In our survey, one-quarter of dentists reported that lack of confidence to critically appraise evidence from scientific journal articles was a barrier to the translation of evidence into practice. Whether Brazilian dentists receive any formal instruction in evidence-based dentistry while in dental school, and how much they are trained in clinical decision-making based on critical appraisal of the scientific literature, should be investigated in future studies.

Interestingly, in another study with Brazilian dentists about the use of scientific evidence in dental practice [7], case reports were the preferred source of information among professionals who claimed to routinely read articles published in scientific journals. Although case reports have an important role in the recognition and description of new diseases or rare manifestations of diseases, study of mechanisms of diseases, and detection of drug side effects [28], they have been placed at the base of the evidence pyramid; their value in facilitating sound clinical decision-making is undermined due to their weaker study design and limited external validity [29].

Taken together, these findings suggest that availability of affordable preappraised, regularly updated, high-quality scientific evidence in the Portuguese language as well as training in the use of the 6S model to search for evidence (i.e., systems, summaries, synopses of syntheses, syntheses, synopses of studies, and studies, beginning the search in the highest possible layer in the model) might help to reduce important barriers to KTE encountered by Brazilian dentists [30].

Additionally, the high popularity of traditional methods for the acquisition of scientific information (i.e., consulting with colleagues and textbooks) observed in our study has also been previously reported in studies performed in other countries (e.g., England, India, Iran, Turkey, and the USA) [11–14,31].

Experts and colleagues are a quick, cheap, and easy to use source of information which offer psychological benefits that journals and online databases cannot offer [32]. Qualitative research has shown that dentists are more inclined to trust what they learn from “real world” clinical experience (i.e., tangible evidence) than what they hear from nonclinical dental academics or read in scientific journals (i.e., intangible evidence) [33].

It is reasonable to expect that seeking advice from experienced and trusted colleagues may help a dentist to determine whether existing scientific evidence is relevant to his/her patient and that expert opinion may provide valuable guidance when there is no conclusive scientific evidence on the best way to solve a given clinical problem. Nevertheless, one cannot guarantee that the colleague being consulted is more knowledgeable than the dentist asking questions [34]. Skills that enable one to perform well in a domain are often the same skills necessary to be able to recognize good performance in that domain; the incompetence of the unskilled usually robs them of the metacognitive ability to realize it (i.e., Dunning–Kruger Effect). Luckily, proper training may improve metacognitive skills [35]. This is another reason for investigating evidence-based dentistry (EBD) instruction throughout dental education. If dental schools do not include EBD in their curricula how will Brazilian dentists become aware of its importance?

In the past decade, a couple of innovative initiatives have been implemented, notably in the USA and the UK, to bridge the gap between research and practice and overcome dentists’ mistrust in researchers and clinical trials. In the USA, the National Institute of Dental and Craniofacial Research, National Institutes of Health, successfully funded a number of projects incorporating clinicians into research groups lead by academics in Dental Practice-Based Research Networks (DPBRN) [36]. DPBRN practitioners are offered several venues to interact with each other (e.g., training meetings, study clubs, participating in webinars, and electronic publications which highlight study results), and research has shown that practitioners’ participation in DPBRN can speed up the translation of research findings into practice [37]. In the United Kingdom, the Collaborations for Leadership in Applied Health Research and Care (CLAHRCs), partnerships between the universities and National Health Service (NHS) Trusts, were developed with the objective of conducting applied health research and translating its findings into day-to-day clinical practice [38]. The latter was based on the concept of communities of practice (CoPs), networks that provide support for formal and informal interaction between novices and experts for learning and sharing knowledge and foster the sense of belonging among members [39]. CoPs may contribute to promoting KTE activities, and the use of a web-based environment for the implementation of CoPs could provide geographically dispersed clinicians with the means to network and communicate more frequently, reducing professional isolation [40]. The extent to which these strategies can be successfully applied to the Brazilian context merits consideration in future studies.

Textbooks can be useful as the backbone to understanding health problems and diseases. However, the time lag between the publication of research showing beneficial or harmful effects of a given clinical intervention and the incorporation of its results into the text of dental books may turn textbooks into outdated or incomplete sources of scientific information for clinical decision-making [41,42]. Therefore, clinicians should be aware that in order to keep pace with new evidence that may be relevant to the provision of high-quality care to their patients, they need to turn to the most appropriate resources such as the Cochrane Library, which publishes and regularly updates systematic reviews of clinical trials [43] and EBD secondary sources focusing on critical summaries of research (e.g, the American Dental Association Evidence-Based Dentistry Website, ADA-EBD, and The Journal of Evidence Based Dentistry and Evidence-Based Dentistry journals) [44,45].

To our great disappointment, search engines (e.g., Google®, Yahoo®, and Bing®) were more frequently used as resources for obtaining scientific information than the Cochrane Library. However, dentists who held an academic position were significantly more likely to search for evidence in the Cochrane Library than those who did not. Thus, it is possible that some close interaction between clinicians and academics, through participation in university-based DPBRN, for example, could benefit clinicians by raising their awareness of the relevance of Cochrane systematic reviews to dental practice and enhancing their adherence to evidence-based treatments [37,46]. This hypothesis should be further investigated.

Online resources contribute to efficient access to both high- and poor-quality scientific publications. Identifying the scientifically meritorious work that directly applies to their patients is the societal responsibility of health professionals [2]. This can be a complex challenge since research has shown that the quality of health-related information on the Internet is often low [47]; even websites of dentists [48,49] and professional associations [50] may not be fully trustworthy. Currently, there are more than a dozen tools (e.g., the Health on the Net, HON code, the Electronic health, eHealth code, and the Completeness, Accuracy, Relevance, and Timeliness tool, CART) to evaluate the trustworthiness of web-based health compendia specifically developed to deliver rapidly accessible evidence-based information (and guidance) to clinicians but to date, there is no standard, validated tool [51].

Social media emerged between 1999 and 2006. Facebook was launched in 2004, YouTube in 2005, and Twitter in 2006. Practicing dentists and consultants began to use these platforms within 2 years of their creation [52]. The virtual environment in which social media has thrived is characterized by the collaborative work of various content creators [53], and many dentists consider technologies such as Facebook®, Instagram® and YouTube® exciting opportunities for marketing and knowledge creation [53,54]. Research has shown that dentists use social media to share clinical and other information with practicing colleagues [52]. For health care organizations, social media has become an important information dissemination tool. However, commentaries to health care-related posts often rely on anecdotes, instead of data from reputable sources, resulting in a large potential for misinformation to spread via social media [55]. Searching for health information on social media also involves the risk of confirmation bias by means of selective exposure to information that confirms one’s existing beliefs and a biased evaluation of this information. In that sense, belief-confirming information may be perceived as being more credible and useful, regardless of its scientific basis [56]. Taking these aspects into account, it was very reassuring to find out that Brazilian dentists do not tend to rely on social media platforms to acquire scientific evidence to inform their clinical practice. However, we cannot rule out the possibility that our findings may have been influenced by social desirability bias [57] or selection bias, especially because one-third of dentists in our sample had an academic position. On the other hand, considering that there is a pressing need for increasing scientific evidence availability in the Portuguese language and that 66% of the Brazilian population has Internet access [58], dental education organizations should consider providing high-quality scientific information on their websites, preferably using well-designed visual aids that may enhance effective communication [59].

Our survey provides relevant information for the improvement of KTE in dentistry, especially in the Brazilian context. Nevertheless, the generalization of our findings to all dentists in Brazil should be made with great caution. Considering that there were 309,088 dentists registered at the National Council of Dentistry (CFO) in August, 2018 [60], less than 1% of the dentists working in the country completed our survey. Moreover, our sample was not a random sample since we were not able to guarantee that every member of the population of Brazilian dentists had an equal and independent chance of selection. For example, dentists who have a postgraduate degree, have an active e-mail account or seek oral health-related information on Facebook® and Instagram® were more likely to be selected than those without these attributes.

It is also worth noting that the distribution of dentists in the Brazil is highly heterogeneous; the ratio of inhabitants/dentists varies from 601 in the Southeast to 1,800 in the North. Southeastern Brazil concentrates 74% of dentists and Rio de Janeiro and São Paulo are the states with the highest number of dentists in the country. Moreover, the Brazilian dental workforce is predominantly female (55%) [61]. In our study most participants were female from the Southeast region of Brazil; however, the percentage distribution of these characteristics in our sample was not similar to their distribution in the overall population of Brazilian dentists. Furthermore, the high rate of respondents with an academic position (31%) may have biased our results towards a higher frequency of use of scientific articles and high-quality evidence (i.e., Cochrane reviews) in clinical decision-making.

## Conclusions

Dentists in our sample showed a positive attitude towards obtaining scientific evidence from reliable sources. However, they reported that there still remain important barriers to the translation of evidence into practice. This can have significant implications for quality of care and should be further investigated.

## Supporting information

S1 Checklist STROBE (Strengthening the reporting of observational studies in epidemiology) checklist. (PDF)

S1 Appendix Questionnaire used for data collection in the original language (Portuguese). (PDF)

S2 Appendix English translation of the questionnaire used for data collection. (PDF)
