# Supplementary material for: Information-seeking behaviors and barriers to the incorporation of scientific evidence into clinical practice: A survey with Brazilian dentists
Source: PLoS One. 2021 Mar 25;16(3):e0249260. doi: 10.1371/journal.pone.0249260 (PMC7993878; doi:10.1371/journal.pone.0249260)
Supplement: S1 Appendix — (PDF) [file pone.0249260.s002.pdf]

Pesquisa - Prática odontológica baseada em evidências científicas

Prezado colega,
Esta pesquisa tem por objetivo fornecer subsídios para a elaboração de estratégias de comunicação que ajudem cirurgiões-dentistas a obter informações para a tomada de decisão clínica baseada em evidências científicas.

Ela tem a aprovação do Comitê de Ética em Pesquisa do Hospital Universitário Pedro Ernesto da Universidade do Estado do Rio de Janeiro (CAAE 94336518.9.0000.5259) e conta com o apoio da Universidade de São Paulo e da Latin American Oral Health Association - LAOHA que é uma associação sem fins lucrativos, de caráter educacional e científico.

O público-alvo são cirurgiões-dentistas de qualquer sexo ou idade e com qualquer tempo de formado, residentes no Brasil.

O preenchimento do questionário leva entre 5 e 10 minutos.

Não existe no questionário qualquer pergunta que permita revelar a identidade dos respondentes. Caso você tenha interesse em obter mais informação sobre a pesquisa ou conhecer os seus resultados envie um email para ebd.laoha@gmail.com .

Importante: não se trata de um teste de conhecimentos; estamos interessados em conhecer as práticas e atitudes dos cirurgiões-dentistas com relação aos temas que estamos pesquisando, de uma forma geral.

Agradecemos a sua valiosa participação.

Branca Heloisa de Oliveira (UERJ), Claudio Pannuti (FOUSP) e Zilson Malheiros (LAOHA)
Coordenadores * Obrigatório

1. Estou participando desta pesquisa voluntariamente e concordo com o uso das informações coletadas pelos pesquisadores, através deste questionário, em eventos e publicações técnico-científicas. *

PARA RESPONDER O QUESTIONÁRIO E AUTORIZAR O USO DE SUAS RESPOSTAS EM EVENTOS E PUBLICAÇÕES TÉCNICO-CIENTÍFICAS É PRECISO QUE VOCÊ SELECIONE "CONCORDO" NAS OPÇÕES ABAIXO.

Marque a opção que se aplica.

- ❑ Concordo
- ❑ Não concordo

INFORMAÇÕES PESSOAIS E PROFISSIONAIS

INICIALMENTE VAMOS PEDIR QUE VOCÊ FORNEÇA ALGUNS DADOS SOBRE VOCÊ E SOBRE A SUA ATIVIDADE PROFISSIONAL NA ODONTOLOGIA.

2. Em qual estado você reside **Marque apenas uma opção.*

⬭ Acre

⬭ Alagoas

⬭ Amapá

⬭ Amazonas

⬭ Bahia

⬭ Ceará

⬭ Federal District

⬭ Espírito Santo

⬭ Goiás

⬭ Maranhão

⬭ Mato Grosso

⬭ Mato Grosso do Sul

⬭ Minas Gerais

⬭ Pará

⬭ Paraíba

⬭ Paraná

⬭ Pernambuco

○ Piauí

○ Rio de Janeiro

○ Rio Grande do Norte

○ Rio Grande do Sul

○ Rondônia

○ Roraima

○ Santa Catarina

○ São Paulo

○ Sergipe

○ Tocantins

3. Qual o ano do seu nascimento? *_Marque apenas uma opção._

○ 1933

○ 1934

○ 1935

○ 1936

○ 1937

○ 1938

○ 1939

○ 1940

○ 1941

○ 1942

○ 1943

○ 1944

○ 1945

○ 1946

○ 1947

○ 1948

○ 1949

○ 1950

○ 1952

○ 1952

○ 1953

○ 1954

○ 1955

○ 1956

○ 1957

○ 1958

○ 1959

○ 1960

○ 1962

○ 1962

○ 1963

○ 1964

○ 1965

○ 1966

○ 1967

○ 1968

○ 1969

○ 1970

○ 1972

○ 1972

○ 1973

○ 1974

○ 1975

○ 1976

○ 1977

○ 1978

○ 1979

○ 1980

○ 1981

○ 1982

○ 1983

○ 1984

○ 1985

○ 1986

○ 1987

○ 1988

○ 1989

○ 1990

○ 1991

○ 1992

○ 1993

○ 1994

○ 1995

○ 1996

○ 1997

○ 1998

○ 1999

○ 2000

○ 2001

○ 2002

4. Qual o seu sexo? **Marque apenas uma opção.*

○ Feminino

○ Masculino

○ Prefiro não informar

5. Em que ano você concluiu o curso de graduação em Odontologia? **Marque apenas uma opção.*

○ 1939

○1940

○1941

○ 1942

○ 1943

○ 1944

- ○ 1945
- ○ 1946
- ○ 1947
- ○ 1948
- ○ 1949
- ○ 1950
- ○ 1952
- ○ 1952
- ○ 1953
- ○ 1954
- ○ 1955
- ○ 1956
- ○ 1957
- ○ 1958
- ○ 1959
- ○ 1960
- ○ 1962
- ○ 1962
- ○ 1963
- ○ 1964
- ○ 1965
- ○ 1966
- ○ 1967
- ○ 1968
- ○ 1969
- ○ 1970
- ○ 1972
- ○ 1972
- ○ 1973
- ○ 1974
- ○ 1975
- ○ 1976
- ○ 1977
- ○ 1978
- ○ 1979
- ○ 1980
- ○ 1981
- ○ 1982
- ○ 1983
- ○ 1984
- ○ 1985
- ○ 1986
- ○ 1987
- ○ 1988
- ○ 1989
- ○ 1990
- ○ 1991
- ○ 1992
- ○ 1993
- ○ 1994
- ○ 1995
- ○ 1996
- ○ 1997
- ○ 1998
- ○ 1999
- ○ 2000
- ○ 2001
- ○ 2002
- ○ 2003
- ○ 2004
- ○ 2005
- ○ 2006
- ○ 2007
- ○ 2008
- ○ 2009
- ○ 2010
- ○ 2011
- ○ 2012
- ○ 2013

⬭ 2014 ⬭ 2016 ⬭ 2018

⬭ 2015 ⬭ 2017

6. Você está envolvido em atividade clínica com paciente, seja direta (em que você realiza o atendimento) ou indiretamente (em que você supervisiona o atendimento feito por outro profissional ou estudante)? **Marque apenas uma opção.*

⬭ Sim, estou envolvido direta ou indiretamente em atividade clínica com paciente.

⬭ Não estou envolvido, direta ou indiretamente, em nenhuma atividade clínica com paciente. **Avance para pergunta 9*

INFORMAÇÕES PROFISSIONAIS

7. Você atua como especialista no atendimento direto ou indireto a pacientes?

* *Marque apenas uma opção.*

⬭ Sim, atuo apenas como especialista.

⬭ Sim, atuo como especialista e como generalista.

⬭ Não.

8. Onde você exerce a sua atividade profissional? *
* Você pode selecionar mais de uma opção caso seja necessário.

Marque as opções que se aplicam

⬭ Consultórios, clínicas ou hospitais da rede pública de saúde (inclui Forças Armadas, Polícia Militar, Corpo de Bombeiros e serviços mantidos pelos poderes Executivo, Legislativo ou Judiciário).

⬭ Consultório, clínica, hospital ou empresa privada (autônomo ou empregado).

⬭ Instituição filantrópica, associação de classe/sindical ou similar.

⬭ Instituição de Ensino Superior.

⬭ Outro.

PARA OS PROFISSIONAIS QUE TÊM ATIVIDADE CLÍNICA COM PACIENTE, DIRETA OU INDIRETA, TEMOS MAIS ALGUMAS PERGUNTAS.

AS PRÓXIMAS DUAS PERGUNTAS SÃO SOBRE A SUA EXPERIÊNCIA COM A PRÁTICA DA ODONTOLOGIA BASEADA EM EVIDÊNCIA CIENTÍFICA.

1. Nos últimos 12 meses, com que frequência você utilizou os seguintes recursos para embasar as suas decisões OU orientações clínicas?

Escolha abaixo, para cada recurso, uma das seguintes opções de resposta: Nunca, Raramente, Às vezes, Quase sempre ou Sempre.

1a. Artigos em revistas científicas * Marque apenas uma opção.

⬭ Nunca

⬭ Raramente

⬭ Às vezes

⬭ Quase sempre

⬭ Sempre

1b. Diretrizes de prática clínica * Marque apenas uma opção.

⬭ Nunca

⬭ Raramente

⬭ Às vezes

⬭ Quase sempre

⬭ Sempre

1c. Aconselhamento com colegas * Marque apenas uma opção.

○ Nunca

○ Raramente

○ Às vezes

○ Quase sempre

○ Sempre

1d. Livros-texto * Marque apenas uma opção.

○ Nunca

○ Raramente

○ Às vezes

○ Quase sempre

○ Sempre

1e. Biblioteca Cochrane * Marque apenas uma opção.

○ Nunca

○ Raramente

○ Às vezes

○ Quase sempre

○ Sempre

1f. Bases de dados bibliográficos como Medline/PubMed, Embase, Lilacs, etc. * Marque apenas uma opção.

○ Nunca

○ Raramente

○ Às vezes

○ Quase sempre

○ Sempre

1g. Facebook® * Marque apenas uma opção.

○ Nunca

○ Raramente

○ Às vezes

○ Quase sempre

○ Sempre

1h. Instagram® * Marque apenas uma opção.

○ Nunca

○ Raramente

○ Às vezes

○ Quase sempre

○ Sempre

1i. Youtube® * Marque apenas uma opção.

○ Nunca

○ Raramente

○ Às vezes

○ Quase sempre

○ Sempre

1j. Ferramentas de busca na Internet como Google®, Yahoo®, Bing® etc. Marque apenas uma opção.

⬭ Nunca

⬭ Raramente

⬭ Às vezes

⬭ Quase sempre

⬭ Sempre

2. Na literatura científica são descritos vários fatores que dificultam a prática odontológica baseada em evidência científica. Considere a SUA PRÓPRIA EXPERIÊNCIA nos últimos 12 meses e responda: Para você, os fatores listados abaixo dificultam a prática da odontologia baseada em evidências científicas?

Escolha abaixo, para cada fator descrito, uma das seguintes opções de resposta: Discordo totalmente, Discordo, Nem discordo/nem concordo, Concordo ou Concordo totalmente.

2a. Falta de tempo para ler artigos científicos. * Marque apenas uma opção.

⬭ Discordo totalmente

⬭ Discordo

⬭ Nem discordo/ nem concordo

⬭ Concordo

⬭ Concordo totalmente

2b. Custo elevado do acesso a artigos científicos. * Marque apenas uma opção.

⬭ Discordo totalmente

⬭ Discordo

⬭ Nem discordo/ nem concordo

- ○ Concordo
- ○ Concordo totalmente

2c. Dificuldade para compreender artigos publicados no idioma inglês.
* Marque apenas uma opção.

- ○ Discordo totalmente
- ○ Discordo
- ○ Nem discordo/ nem concordo
- ○ Concordo
- ○ Concordo totalmente

2d. Insegurança para analisar criticamente artigos científicos ao ponto de sintetizar e usar os seus resultados na prática clínica.* Marque apenas uma opção.

- ○ Discordo totalmente
- ○ Discordo
- ○ Nem discordo/ nem concordo
- ○ Concordo
- ○ Concordo totalmente

2e. Dificuldade para saber se informações científicas encontradas na Internet são confiáveis.
* Marque apenas uma opção.

- ○ Discordo totalmente
- ○ Discordo
- ○ Nem discordo/ nem concordo
- ○ Concordo
- ○ Concordo totalmente

Agradecemos por sua participação!

9. Caso tenha interesse em receber o resultado desta pesquisa, escreva o seu email abaixo (optativo) ou envie mensagem para ebd.laoha@gmail.com
