# Supplementary material for: Information-seeking behaviors and barriers to the incorporation of scientific evidence into clinical practice: A survey with Brazilian dentists
Source: PLoS One. 2021 Mar 25;16(3):e0249260. doi: 10.1371/journal.pone.0249260 (PMC7993878; doi:10.1371/journal.pone.0249260)
Supplement: S2 Appendix — (PDF) [file pone.0249260.s003.pdf]

Research - Dental practice based on scientific evidence

Dear colleague,

This research aims to provide support for the development of communication strategies that may help dental surgeons to obtain information for clinical decision-making based on scientific evidence.

It has the approval of the Research Ethics Committee of the Pedro Ernesto University Hospital of the Rio de Janeiro State University (CAAE 94336518.9.0000.5259) and has the support of the University of São Paulo and the Latin American Oral Health Association – LAOHA, which is a non-profit educational and scientific organization.

The target audience of this research is Brazilian dentists of any gender, age and degree of training residing in Brazil.

Filling out the questionnaire takes between 5 and 10 minutes.

This survey is anonymous. Therefore, no question will ask you to reveal your identity. However, if you are interested in obtaining more information about the research or if you are willling to know the results of our survey after the data analysis is completed, you may print your e-mail address at the end of the questionnaire or send an e-mail to ebd.laoha@gmail.com .

Important: this is not a test of knowledge; we are interested in knowing the practices and attitudes of dentists in general regarding the subjects under investigation.

We appreciate your valuable contribution.

Branca Heloisa de Oliveira (UERJ), Claudio Pannuti (FOUSP) and Zilson Malheiros (LAOHA) Coordinators of the study “Dental Practice Based on Scientific Evidence” * Required

1. I am participating in this research voluntarily and agree to the use of the information collected by the researchers, through this questionnaire, in technical-scientific events and publications. *

TO ANSWER THE QUESTIONNAIRE AND AUTHORIZE THE USE OF YOUR RESPONSES IN TECHNICAL-SCIENTIFIC EVENTS AND PUBLICATIONS YOU MUST SELECT "I AGREE" IN THE OPTIONS BELOW.

Check all that apply.

- ❑ I Agree
- ❑ Not agree

PERSONAL AND PROFESSIONAL INFORMATION

WE WILL ASK YOU TO PROVIDE SOME DATA ABOUT YOU AND YOUR PROFESSIONAL ACTIVITY IN DENTISTRY.

2. In which Brazilian state do you live? **Mark only one oval.*

⬭ Acre

⬭ Alagoas

⬭ Amapá

⬭ Amazonas

⬭ Bahia

⬭ Ceará

⬭ Federal District

⬭ Espírito Santo

⬭ Goiás

⬭ Maranhão

⬭ Mato Grosso

⬭ Mato Grosso do Sul

⬭ Minas Gerais

⬭ Pará

⬭ Paraíba

⬭ Paraná

⬭ Pernambuco

⬭ Piauí

⬭ Rio de Janeiro

⬭ Rio Grande do Norte

⬭ Rio Grande do Sul

⬭ Rondônia

⬭ Roraima

⬭ Santa Catarina

⬭ São Paulo

⬭ Sergipe

⬭ Tocantins

3. In which year were you born? * *Mark only one oval.*

⬭ 1933

⬭ 1934

⬭ 1935

⬭ 1936

⬭ 1937

⬭ 1938

⬭ 1939

⬭ 1940

⬭ 1941

⬭ 1942

⬭ 1943

⬭ 1944

⬭ 1945

⬭ 1946

⬭ 1947

⬭ 1948

⬭ 1949

⬭ 1950

⬭ 1952

⬭ 1952

⬭ 1953

⬭ 1954

⬭ 1955

- ⬭ 1956
- ⬭ 1957
- ⬭ 1958
- ⬭ 1959
- ⬭ 1960
- ⬭ 1962
- ⬭ 1962
- ⬭ 1963
- ⬭ 1964
- ⬭ 1965
- ⬭ 1966
- ⬭ 1967
- ⬭ 1968
- ⬭ 1969
- ⬭ 1970
- ⬭ 1972
- ⬭ 1972
- ⬭ 1973
- ⬭ 1974
- ⬭ 1975
- ⬭ 1976
- ⬭ 1977
- ⬭ 1978
- ⬭ 1979
- ⬭ 1980
- ⬭ 1981
- ⬭ 1982
- ⬭ 1983
- ⬭ 1984
- ⬭ 1985
- ⬭ 1986
- ⬭ 1987
- ⬭ 1988
- ⬭ 1989
- ⬭ 1990
- ⬭ 1991
- ⬭ 1992
- ⬭ 1993
- ⬭ 1994
- ⬭ 1995
- ⬭ 1996
- ⬭ 1997
- ⬭ 1998
- ⬭ 1999
- ⬭ 2000
- ⬭ 2001
- ⬭ 2002

4. Which is your gender? **Mark only one oval.*

- ⬭ Female
- ⬭ Male
- ⬭ I prefer not to answer

5. In which year did you complete the Dentistry undergraduate course?
**Mark only one oval.*

○ 1939
○1940
○1941
○ 1942
○ 1943
○ 1944
○ 1945
○ 1946
○ 1947
○ 1948
○ 1949
○ 1950
○ 1952
○ 1952
○ 1953
○ 1954
○ 1955
○ 1956
○ 1957
○ 1958
○ 1959
○ 1960
○ 1962
○ 1962
○ 1963
○ 1964
○ 1965
○ 1966
○ 1967
○ 1968
○ 1969
○ 1970
○ 1972
○ 1972
○ 1973
○ 1974
○ 1975
○ 1976
○ 1977
○ 1978
○ 1979
○ 1980
○ 1981
○ 1982
○ 1983
○ 1984
○ 1985
○ 1986
○ 1987
○ 1988
○ 1989
○ 1990
○ 1991
○ 1992
○ 1993
○ 1994
○ 1995
○ 1996
○ 1997
○ 1998
○ 1999
○ 2000
○ 2001

○ 2002
○ 2003
○ 2004
○ 2005
○ 2006
○ 2007
○ 2008
○ 2009
○ 2010
○ 2011
○ 2012
○ 2013
○ 2014
○ 2015
○ 2016
○ 2017
○ 2018

6. Are you involved in clinical activity with patients directly (in which you perform dental procedures yourself dental service) or indirectly (in which you supervise the work performed by another dental professional or a dental student)? *

Mark only one oval.

○ Yes, I am involved directly or indirectly in clinical activity with a patient.

○ I am not involved, directly or indirectly, in any clinical activity with a patient. **Skip to question 9*

PROFESSIONAL INFORMATION

7. Do you act as a specialist in direct or indirect patient care?

* *Mark only one oval.*

○ Yes, I act only as a specialist.

○ Yes, I work as a specialist and as a generalist.

○ No.

8. Where do you exercise your professional activity? *
* You can select more than one option if necessary.

Check all that apply.

⬭ Public health clinics, clinics or hospitals (includes Armed Forces, Military Police, Fire Department and services maintained by the Executive, Legislative or Judicial branches).

⬭ Office, clinic, hospital or private company (self-employed or employed).

⬭ Philanthropic institution, trade / union association or similar.

⬭ Higher Education Institution.

⬭ Other

FOR PROFESSIONALS WHO HAVE CLINICAL ACTIVITY WITH A PATIENT, DIRECT OR INDIRECT, WE HAVE A FEW MORE QUESTIONS.

THE NEXT TWO QUESTIONS ARE ABOUT YOUR EXPERIENCE WITH THE PRACTICE OF DENTISTRY BASED ON SCIENTIFIC EVIDENCE

1. In the past 12 months, how often have you used the following resources to support your clinical decisions OR those of the dentists/students whom you supervise?

Choose below, for each question, one of the following response options: Never, Hardly ever, Occasionally, Often or Very often.

1a. Scientific articles * Mark only one oval

⬭ Never

⬭ Hardly ever

⬭ Occasionally

⬭ Often

⬭ Very often

1b. Clinical guidelines * Mark only one oval

⬭ Never

⬭ Hardly ever

- Occasionally
- Often
- Very often

1c. Counseling with colleagues * Mark only one oval

- Never
- Hardly ever
- Occasionally
- Often
- Very often

1d. Textbooks * Mark only one oval

- Never
- Hardly ever
- Occasionally
- Often
- Very often

1e. The Cochrane Library * Mark only one oval

- Never
- Hardly ever
- Occasionally
- Often
- Very often

1f. Bibliographic databases such as MEDLINE/PubMed, Embase, Lilacs, etc. * Mark only one oval

- ⬭ Never
- ⬭ Hardly ever
- ⬭ Occasionally
- ⬭ Often
- ⬭ Very often

1g. Facebook® * Mark only one oval

- ⬭ Never
- ⬭ Hardly ever
- ⬭ Occasionally
- ⬭ Often
- ⬭ Very often

1h. Instagram® * Mark only one oval

- ⬭ Never
- ⬭ Hardly ever
- ⬭ Occasionally
- ⬭ Often
- ⬭ Very often

1i. Youtube® * Mark only one oval

- ⬭ Never
- ⬭ Hardly ever

⬭ Occasionally

⬭ Often

⬭ Very often

1j. Search engines such as Google®, Yahoo®, Bing® etc.* Mark only one oval

⬭ Never

⬭ Hardly ever

⬭ Occasionally

⬭ Often

⬭ Very often

2. In the scientific literature, there are several factors that have been reported as hindrances to dental practice based on scientific evidence.
Consider YOUR OWN EXPERIENCE in the last 12 months and answer:

For you, the factors listed below make it difficult to practice dentistry based on scientific evidence?

Choose below, for each factor described, one of the following response options: Strongly disagree, Disagree, Neither agree / nor disagree, Agree or Strongly Agree.

2a. Lack of time to read scientific articles. * *Mark only one oval.*

⬭ Strongly disagree

⬭ Disagree

⬭ Neither agree / nor disagree

⬭ Agree

⬭ Strongly Agree

2b. High cost charged fot the access to scientific articles. * Mark only one oval

- ⬭ Strongly disagree
- ⬭ Disagree
- ⬭ Neither agree / nor disagree
- ⬭ Agree
- ⬭ Strongly Agree

2c. Difficulty understanding articles published in the English language.
* Mark only one oval

- ⬭ Strongly disagree
- ⬭ Disagree
- ⬭ Neither agree / nor disagree
- ⬭ Agree
- ⬭ Strongly Agree

2d. Dificultty in critically analyzing scientific articles to the point of synthesizing and using their results in clinical practice. * Mark only one oval

- ⬭ Strongly disagree
- ⬭ Disagree
- ⬭ Neither agree / nor disagree
- ⬭ Agree
- ⬭ Strongly Agree

2e. Difficulty in knowing whether scientific information found on the Internet is reliable.
* Mark only one oval

- ⬭ Strongly disagree
- ⬭ Disagree

- Neither agree / nor disagree
- Agree
- Strongly Agree

Thank you for your participation!

9- If you are interested in receiving the results of this research, write your email below (optional) or send a message to ebd.laoha@gmail.com
